# Electrical performance of lightweight CNT-Cu composite wires impacted by surface and internal Cu spatial distribution

**DOI:** 10.1038/s41598-017-09279-x

**Published:** 2017-08-24

**Authors:** Rajyashree Sundaram, Takeo Yamada, Kenji Hata, Atsuko Sekiguchi

**Affiliations:** 1 Technology Research Association for Single Wall Carbon Nanotubes (TASC), Central 5, 1-1-1 Higashi, Tsukuba, 305-8565 Japan; 2 National Institute of Advanced Industrial Science and Technology (AIST), Central 5, 1-1-1 Higashi, Tsukuba, 305-8565 Japan; 3 Present Address: National Institute of Advanced Industrial Science and Technology (AIST), Central 5, 1-1-1 Higashi, Tsukuba, 305-8565 Japan

## Abstract

We report ultralong conducting lightweight multiwall carbon nanotube (MWCNT)-Cu composite wires with MWCNTs uniformly distributed in a continuous Cu matrix throughout. With a high MWCNT vol% (40–45%), the MWCNT-Cu wire density was 2/3^rd^ that of Cu. Our composite wires show manufacturing potential because we used industrially compatible Cu electrodeposition protocols on commercial CNT wires. Further, we systematically varied Cu spatial distribution on the composite wire surface and bulk and measured the associated electrical performance, including resistivity (ρ), temperature dependence of resistance, and stability to current (measured as current carrying capacity, CCC in vacuum). We find that a continuous Cu matrix with homogeneous MWCNT distribution, i.e., maximum internal Cu filling within MWCNT wires, is critical to high overall electrical performances. Wires with maximum internal Cu filling exhibit (i) low room temperature ρ, 1/100th of the starting MWCNT wires, (ii) suppressed resistance-rise with temperature-increase and temperature coefficient of resistance (TCR) ½ that of Cu, and (iii) vacuum-CCC 28% higher than Cu. Further, the wires showed real-world applicability and were easily soldered into practical circuits. Hence, our MWCNT-Cu wires are promising lightweight alternatives to Cu wiring for weight-reducing applications. The low TCR is specifically advantageous for stable high-temperature operation, e.g., in motor windings.

## Introduction

Substituting Cu wires with a lighter material in aerospace and automobile applications is expected to immensely impact fuel savings and reduce CO_2_ emissions. Hence, the demand for robust lightweight conducting wires is rapidly rising. One strategy to cater to this demand is developing high-performance Cu-matrix composites containing weight-reducing nanocarbon fillers with remarkable properties, such as CNTs. CNT-Cu composites have already demonstrated electrical^1–3^, thermal^3–5^, and mechanical properties^6–13^ rivalling that of Cu. Specifically, composites of aligned CNTs uniformly embedded in a continuous Cu matrix have shown electrical conductivities similar to and exceeding Cu at room temperature and high temperature, respectively and maximum current carrying capacities (CCC) 100-fold higher than Cu in vacuum^1–3^. Calculations indicate that CNT addition increases Cu diffusion activation energy, curbing Cu failure in the composites^1^. However, preparing such CNT-Cu composites as macroscopic wires with high electrical performance and scalability potential is challenging and has not been reported yet.

CNT-Cu composites can be fabricated by two routes, (i) powder-based mixing and compaction of CNTs and Cu^5–13^ or (ii) Cu electrodeposition on/into CNT assemblies^1–4, 14–18^. Powder-based mixing and compaction often employs easily scalable routine metallurgical processes, such as ball milling, sintering, etc. However, it is difficult to prevent defect-inclusion in nanotubes and control CNT content and alignment in CNT-Cu composites prepared by powder-based methods. Harsh conditions used for both mixing (ultrasonication^9^, ball milling^10–13^, etc.) and compaction (sintering^5–11^, high-pressure compression and torsion^12, 13^) can damage CNTs^11^. Further, due to limited wettability of CNTs by Cu, mixing and compaction often results in inhomogeneous CNT distribution in the Cu matrix and rampant CNT phase separation and agglomeration, especially at Cu grain boundaries. Consequently, controlling CNT volume fractions, especially at the higher ends is problematic. CNT-Cu composites made by mixing and compaction with >20 vol% CNTs have indeed not been reported so far. Also, the mixing step inherently precludes regulating CNT alignment in the composite and evaluating its effect on CNT-Cu performance. Therefore, mixing and compaction hampers systematically varying CNT-Cu structure and drawing reliable structure-property correlations, undermining gaining insight into the true potential of CNT-Cu composites.

On the other hand, electrochemical Cu deposition of CNT wires is benign and affords flexibility to controllably vary composite composition and CNT alignment. Production of CNT wires with tailored internal structures is well-known^19–21^ and these materials are even available commercially^22–24^. Cu electrodeposition has been previously applied to CNT assemblies, especially wires synthesized at labscale^14–18^. Nevertheless, only Cu-coated CNT wires with little or non-uniform Cu penetration within have been obtained so far due to the use of typical aqueous solution-based Cu electrodeposition on hydrophobic CNT starting materials^14–16^. Shuai *et al*.^17^ and Jin *et al*.^18^ prepared CNT-Cu sheets with high internal Cu penetration by repeated Cu electrodeposition into superaligned MWCNT sheets from aqueous CuSO_4_/H_2_SO_4_ solutions containing glucose. However, their composites contain low CNT fractions of ~1-5 vol%. Subramaniam *et al*. devised a novel 2-step electrodeposition process to fabricate CNT-Cu composites with complete internal Cu filling and high CNT fractions ranging to 45 vol%^1^. To enable Cu penetration within nanotube starting materials, first, Cu seeds were electrodeposited from organic solutions of Cu salts that wet hydrophobic CNT assemblies. Subsequently, the Cu seeds were grown using conventional CuSO_4_-based aqueous deposition to achieve Cu filling in the second step. By this method, only microscale structures and small sheets were fabricated^1–4^. There are no reports on the fabrication of several cm long CNT-Cu composite wires with high CNT volume fractions and complete internal Cu filling throughout by electrodeposition. In addition, to the best of our knowledge, there are no studies that correlate the comprehensive CNT-Cu electrical performance, including resistivity (ρ), its temperature dependence, and CCC with Cu spatial distribution in the composites. The Cu spatial distribution, i.e., Cu content at the composite surface and bulk, is likely to have a marked influence on CNT-Cu properties. All previous studies, irrespective of the fabrication methodology, have focused on CNT-Cu electrical performance, chiefly ρ at room temperature (ρ_RT_) only as a function of the total Cu content measured either as vol% or sample thickness. However, the total Cu content does not reflect the Cu spatial distribution in the composite.

Here, we report the preparation of very long (up to 10 cm) lightweight CNT-Cu composite wires with MWCNTs homogeneously distributed throughout a continuous Cu matrix all along the wire. We used 2-step Cu electrodeposition into industrial MWCNT wires to achieve high internal Cu filling in our composite wires. With 45 vol% CNTs, the density of our MWCNT-Cu wires is 2/3^rd^ that of Cu. The composite wires exhibit promising electrical performances, including low ρ_RT_ (1/100th that of the starting MWCNT wires), lower resistance-change with increasing temperature than Cu, and high CCCs (in vacuum) exceeding that of Cu. Further, we controllably varied the Cu spatial distribution in the CNT-Cu wires and evaluated the corresponding electrical properties. Our results demonstrate that maximizing the Cu content in the bulk to achieve a continuous Cu matrix throughout is vital to overall high electrical performances.

## Results and Discussion

**Figure 1:**
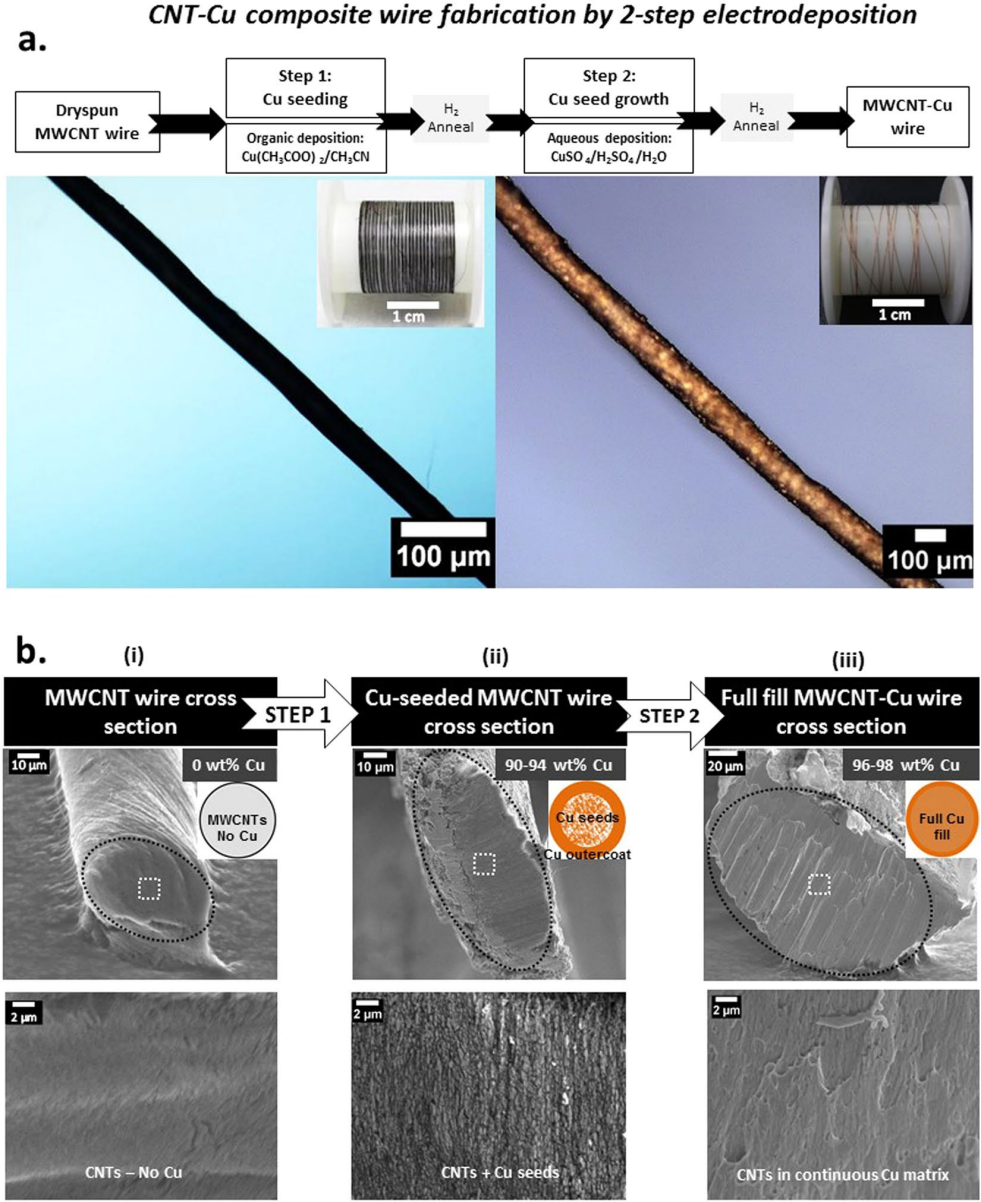
(**a**) CNT-Cu composite wire fabrication by 2-step electrodeposition. (**b**) Cross sectional SEM images of the (i) starting MWCNT wire, (ii) Cu-seeded MWCNT wire after step 1 (deposition in organic electrolyte), and (iii) fully filled MWCNT-Cu wire obtained after step 2 (deposition in aqueous electrolyte). The black dotted ellipsoids in the low-magnification images (top) represent the cross section outlines. The high-magnification images (bottom) are a zoom into the area enclosed by the white dotted-line square in the low-magnification images (top).

We demonstrate the fabrication of lightweight MWCNT-Cu wires with uniform CNT distribution in a continuous Cu matrix throughout the wire length. The continuous Cu matrix in the composite wires was achieved by facilitating maximum (full) internal Cu filling using a novel 2-step electrodeposition into neat MWCNT wires. Figure 1b shows the typical cross section of the fully filled CNT-Cu wires (iii) obtained after optimal Cu seeding (ii) into the starting MWCNT wires (i). The composite wires with full filling contain ~96–98 wt% Cu and show densities ~5.2 g/cc, 2/3^rd^ that of Cu. The SEM cross sections revealed a continuous Cu matrix with very few pores (Fig. 1b, iii). Multiple cross sections analysed (3–5 per cm of the wire) indicate that full Cu filling was achieved throughout the wire length (Supplementary Fig. S1). Up to 10 cm long wires of these CNT-Cu composites (Fig. 1a) could be fabricated from industrial nanotube wire spools, indicating potential fabrication scalability. Our fabrication protocol can potentially produce longer samples and the sample length was limited only by the size of the electrodeposition set-up we used. CNT-Cu wires with lengths ranging from ~50 cm^14^ to a few meters^9, 15^ have been previously reported. However, these wires were either powder-processed composites with a low CNT content (~0.5 vol%) and densities similar to Cu^9^ or only Cu-coated CNT wires (without a continuous Cu matrix)^14, 15^.

**Figure 2:**
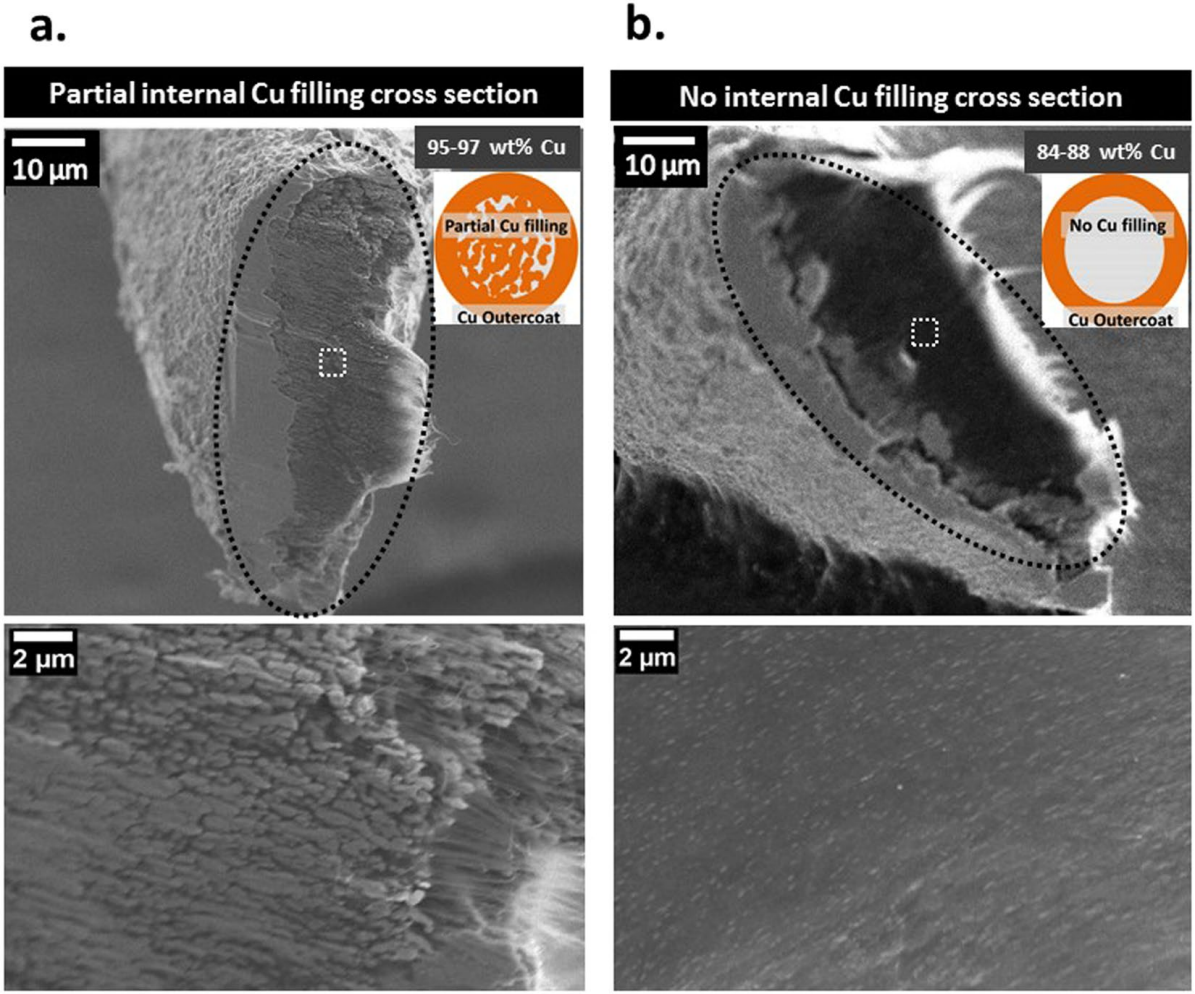
Cross section SEM images of MWCNT-Cu wires with (**a**) partial internal Cu filling and (**b**) only Cu outercoating and no internal Cu filling (core-sheath structure). The black dotted ellipsoids in the low-magnification images (top) represent the cross section outlines. The high-magnification images (bottom) are a zoom into the area enclosed by the white dotted-line square in the low-magnification images (top).

**Figure 3:**
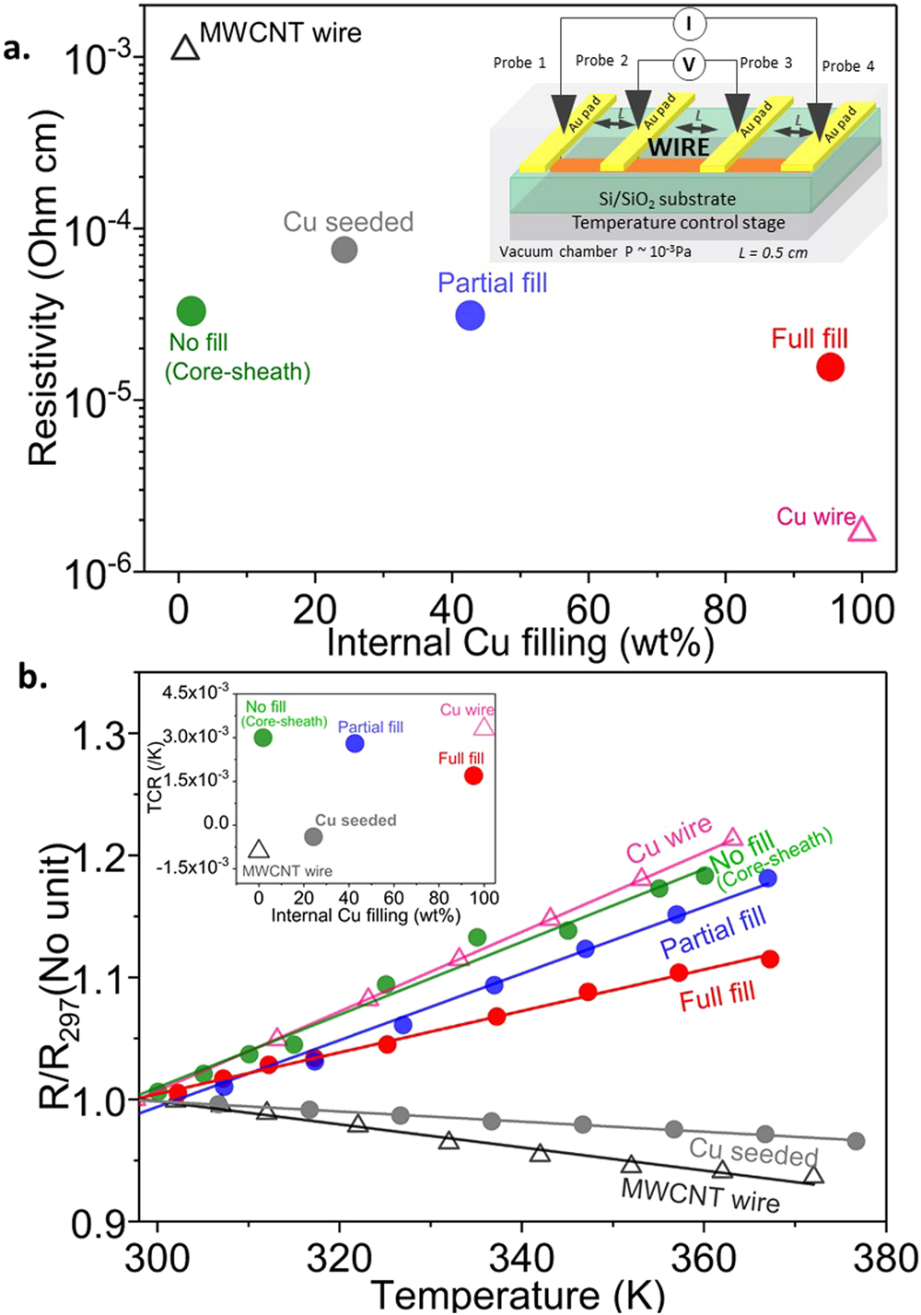
(**a**) Room temperature resistivity and (**b**) temperature dependence of resistance (inset: TCR) of the starting MWCNT wire, MWCNT-Cu wires with various levels of internal filling, and Cu wires. The 4-probe resistance measurement set-up is shown in the inset of **a**.

**Figure 4:**
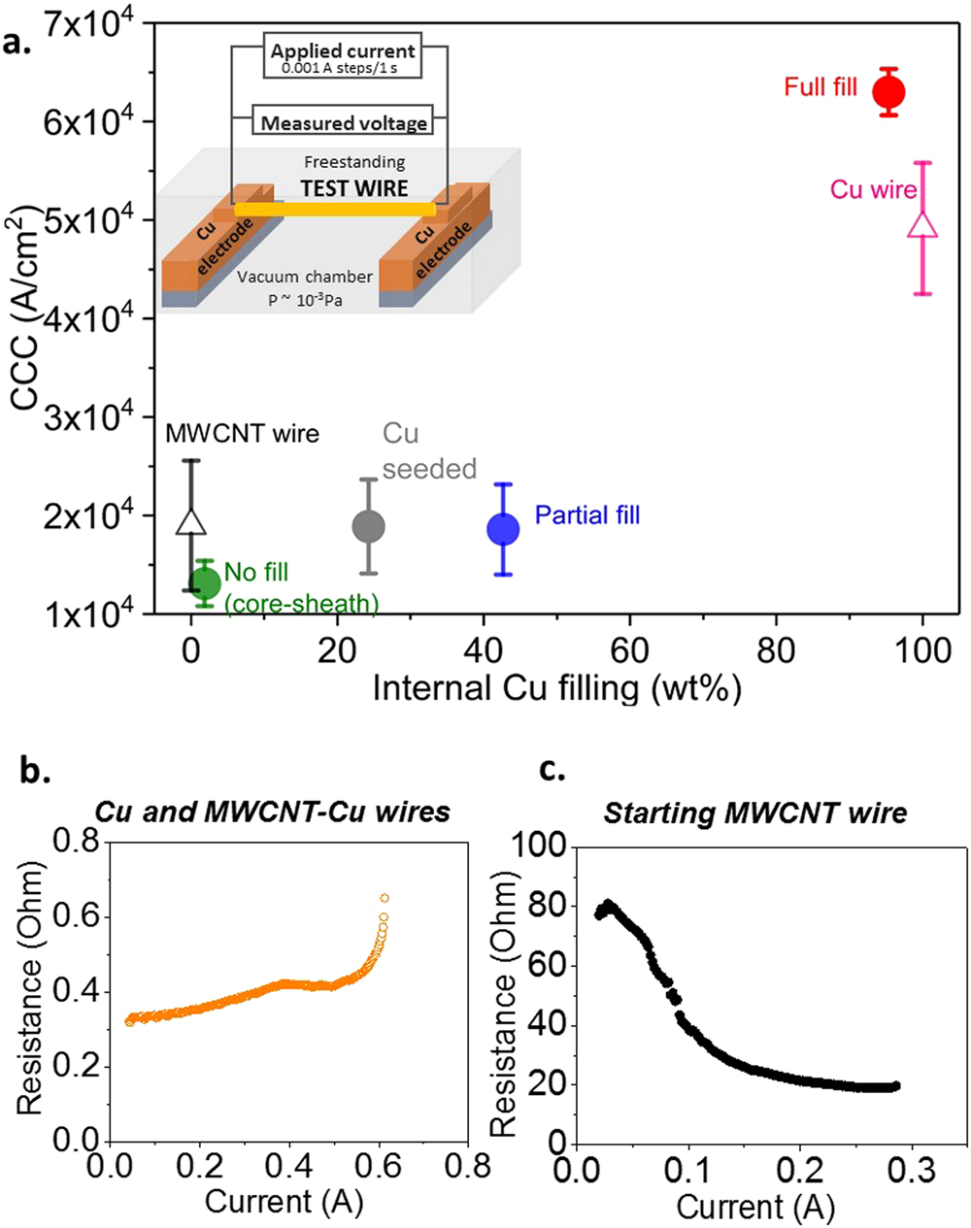
(**a**) CCC of the starting MWCNT wire, MWCNT-Cu wires with various levels of internal Cu filling, and Cu wire. Inset shows the CCC measurement set-up. The typical resistance vs. applied current of the (**b**) conducting Cu/MWCNT-Cu wires and (**c**) starting MWCNT wires.

Electrical characterization data acquired vs. Cu spatial distribution in the CNT-Cu wires demonstrate that maximizing internal Cu filling to achieve a contiguous Cu matrix throughout is vital to achieving favourable overall electrical performance. To the best of our knowledge, this is the first report to systematically correlate overall CNT-Cu composite electrical performance as a function of Cu spatial distribution. In comparison to Cu-seeded wires and those with partial or no internal Cu filling (cross sections in Fig. 2), the fully filled MWCNT-Cu wires show the lowest ρ_RT_ (Fig. 3a). The ρ_RT_ of the MWCNT-Cu wires with full internal filling is typically 1.6 × 10^−5^ Ohm cm, 100 times lower than the starting MWCNT wires. The fully filled composite wires also show suppressed resistance-increase with temperature (Fig. 3b). The temperature coefficient of resistance (TCR), which quantifies the resistance-change with temperature, is ~1.7 × 10^−3^ for the fully filled MWCNT-Cu wire, ~½ that of Cu (Fig. 3b, inset). In comparison, wires with partial/no internal Cu filling show TCRs similar to Cu. This combination of low TCR and low ρ_RT_ is promising for applications requiring stable and efficient high-temperature operation, such as lightweight motor windings. Further, in terms of stability to high currents (Fig. 4a), full Cu internal filling throughout leads to CCC values (in vacuum) 28% higher than Cu wires of similar dimensions and 3 × higher than starting MWCNT wires. In contrast, Cu-seeded wires and samples with no/partial Cu filling show lower vacuum CCC values similar to the starting MWCNT wires.

**Figure 5:**
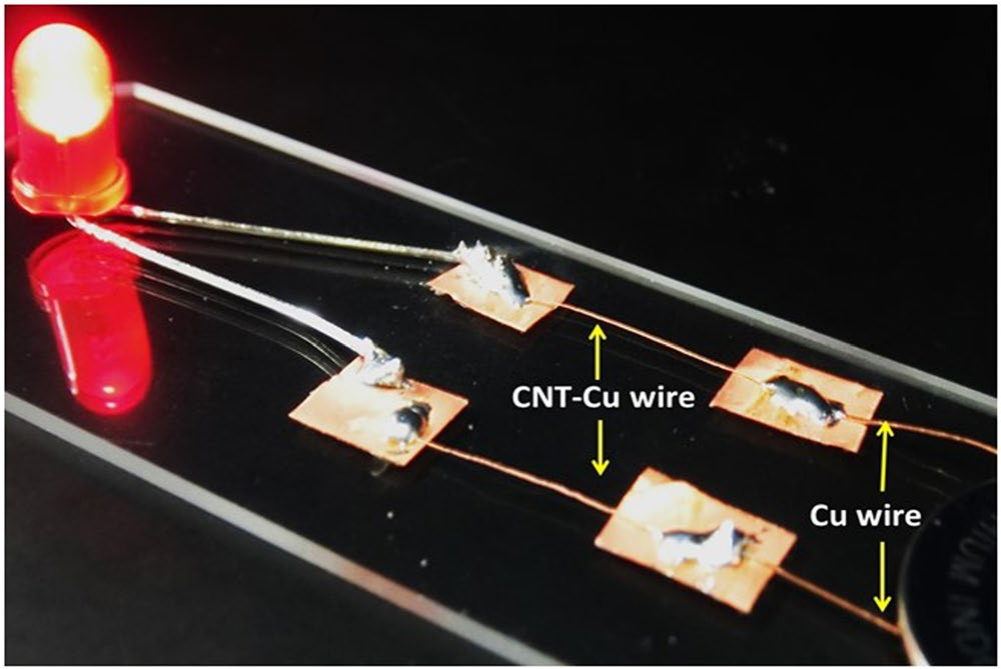
MWCNT-Cu wires in a simple circuit integrated by regular soldering along with Cu wires demonstrating their practical applicability.

In addition, our MWCNT-Cu composite wires could be easily integrated into practical circuits with regular Cu wires by simple soldering, as shown in Fig. 5. Neat CNT wires have been proposed as lightweight conductors replacing metal wires^25^. However, integrating CNT wires into mainstream circuits using Cu is challenging because regular solder does not wet hydrophobic CNT wires, requiring specialized contacting procedures^26^. Our MWCNT-Cu wires, with the combination of light weight, low resistivity, and easy integrability, offer a distinct advantage over neat CNT wires.

To understand the observed variation of CNT-Cu wire electrical performance with Cu spatial distribution in the composite surface and bulk, it is essential to discuss the electrical properties in the context of specific structural differences among the wire samples considered.

### Cross sectional structure of MWCNT-Cu wires vs. internal Cu filling

**Figure 6:**
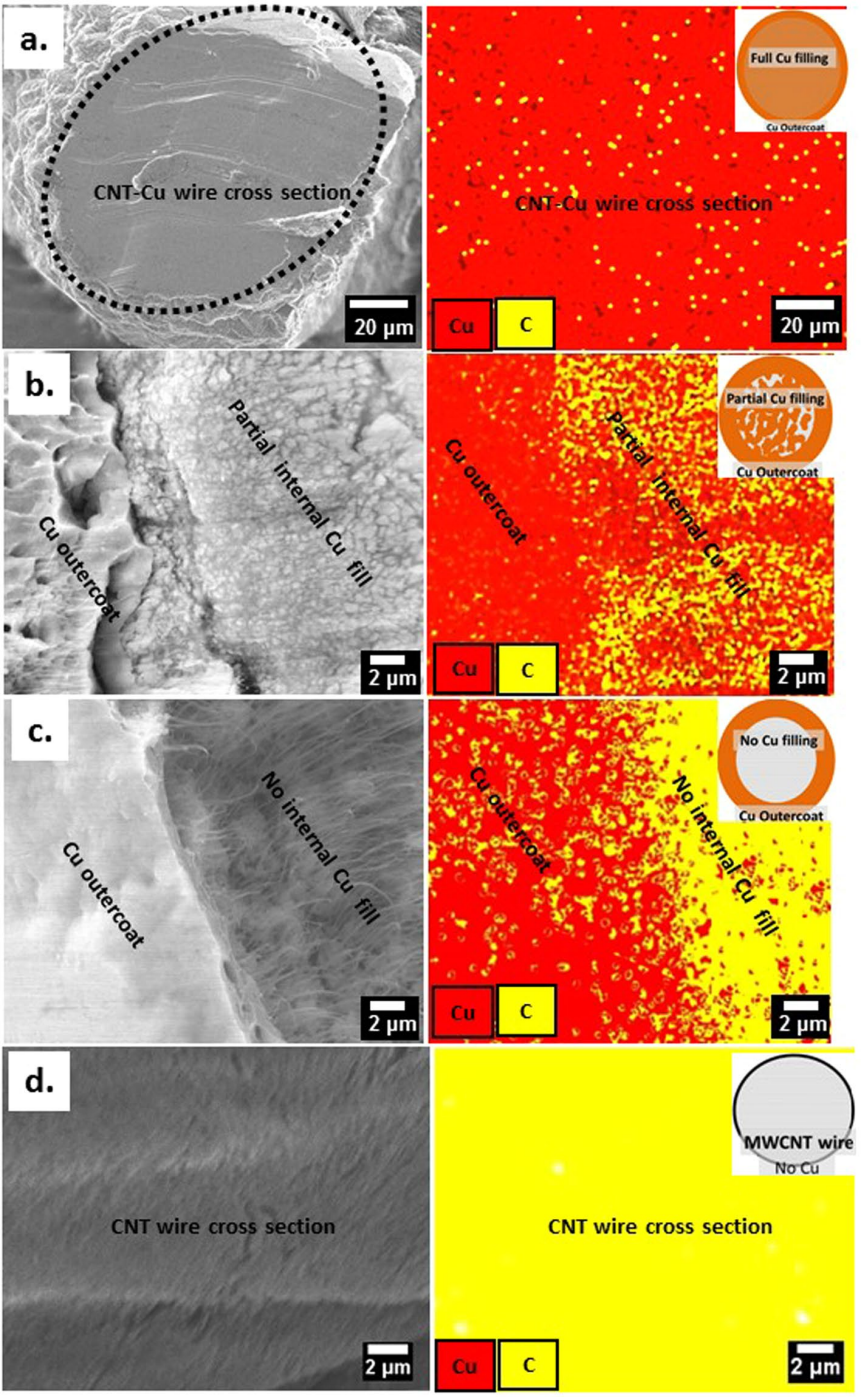
Cross section SEM-EDX maps of MWCNT-Cu wires with (**a**) full, (**b**) partial, and (**c**) no internal Cu filling and (**d**) starting MWCNT wires. The black dotted ellipsoid in a. is the cross section outline.

The total Cu content is high in all types of wires prepared (>85 wt%, Supplementary Fig. S2). However, the amount (Supplementary Fig. S2), size, continuity, and distribution of Cu grains within are distinctly different among the samples (Figs 1b, 2 and 6). As seen from the CS-SEM images (Fig. 1b, ii), Cu-seeded wires contain discontinuously distributed spheroidal small Cu seeds (~200-800 nm in diameter) within. The Cu grains on the outercoating are not always contiguously connected. All wires subjected to CuSO_4_-deposition, irrespective of prior seeding, show the presence of larger Cu grains and a continuous Cu outercoating. In the fully filled samples, the Cu outercoating is indistinguishable from the bulk MWCNT-Cu wires (Fig. 1b, iii). The CNTs are completely coated by Cu, as seen from the scarcity of C signal in the CS-SEM-EDX map (Fig. 6a). Further, hardly any pores/gaps/cracks are observed in the SEM cross sections of the fully filled wires (Fig. 1b iii and Supplementary Fig S1). Therefore, our optimal 2-step electrodeposition protocol resulted in sufficient inclusion of Cu within the MWCNT wires to form composite wires (~90–100 μm in diameter) with a solid continuous Cu matrix. Contrastingly, the partially filled wires consist of numerous 100 s of nm to micron-sized loosely connected Cu grains within, in addition to a distinct Cu outercoating, as seen in the CS-SEM image of Fig. 2a. The EDX map (Fig. 6b) shows the strong presence of both C and Cu signals in the sample bulk as opposed to the dominant presence of only Cu in the outercoating. Wires with no Cu internal filling obtained by direct aqueous CuSO_4_-deposition without prior seeding consist of only a continuous Cu outercoating on the MWCNT wire with very little inside as shown in the CS-SEM image (Fig. 2b, EDX map shown in Fig. 6c). For comparison, the EDX map of the neat MWCNT wire cross section with no Cu signal is provided in Fig. 6d.

We propose that these differences in Cu spatial distribution in the various MWCNT-Cu wires determines the dominant electron conducting component i.e., Cu or MWCNTs, resulting in the variation of ρ_RT_, TCR, and CCC among the samples. Each of these properties is discussed in the sections below.

### Room temperature resistivity, ρ_RT_ of MWNCT-Cu wires vs. internal Cu filling

With addition of metallic Cu by seeding and subsequent aqueous deposition, ρ_RT_ of the MWCNT wires decreases (Fig. 3a and supplementary Table S1). The Cu-seeded MWCNT wires (total Cu content = 90–94 wt%) show higher ρ_RT_ than all the aqueous deposited wires, including wires with no internal Cu filling (only Cu outercoating) with the lowest total Cu content (84–88 wt%). In the Cu-seeded wires, the disconnected small Cu grains act as electron scattering points, resulting in higher ρ_RT_ values. On the other hand, in all the wires subjected to aqueous CuSO_4_ deposition, metallic Cu is present in a continuous form, either as outercoating and/or as internal filling, offering a lower resistive path for electron transport. This leads to reduced ρ_RT_ values for the full, partial, and no internal Cu filling composite wires, in comparison to the Cu-seeded wires. However, among the full, partial, and no internal Cu filling composite wires, the ρ_RT_ values vary because of differences in the Cu spatial distribution. The fully filled MWCNT-Cu wires show the lowest ρ_RT_ among all samples (~1.6 × 10^−5^ Ohm cm), nearly ½ that of wires with no or partial internal Cu filling. We attribute this to the presence of a continuous conducting Cu matrix in the fully filled wires. In contrast, in wires with no or partial internal filling, the CNTs and any disconnected internal Cu grains within contribute to the cross sectional area and sample resistance by increasing electron scattering, leading to higher ρ_RT_. It is noteworthy that ρ_RT_ of wires with only Cu outercoating and no internal filling (core-sheath structures) is similar to previous reports on Cu-coated wires with comparable MWCNT wire diameter to Cu outer coating thickness ratios^14, 15^.

Yet, the ρ_RT_ of the fully filled wires is still an order of magnitude higher than that of Cu despite the presence of a continuous Cu matrix. The MWCNTs, nanotube ends, and CNT/Cu interface can be expected to scatter electrons leading to increased resistance. Nevertheless, we are unable to clearly demonstrate the role of the MWCNT/Cu interface in the electron transport. Comparing with literature, previous CNT-Cu composites with continuous Cu matrices but low CNT fractions (<20 vol%)^8, 9, 16^ show ρ_RT_ values of the same order of magnitude as that of Cu. In contrast, our fully filled MWCNT-Cu wires contain higher CNT content (40–45 vol%), which may contribute to the high observed ρ_RT_ (10 × Cu). Further, most previous low CNT fraction composites were prepared by powder processing and the Cu grain quality is improved during compaction (sintering). However, our MWCNT-Cu wires were not subjected to treatments typical to powder processing^5–13^, such as annealing at temperatures >500 °C, drawing, high-pressure procedures, etc. It is worth noting that the advantage of low ρ_RT_ in the low CNT vol% composites is overturned by their high densities, typically ~8 g/cc^5^, nearly equal to that of Cu (8.9 g/cc). On the other hand, the density of our 45 vol% CNT-Cu composites is ~5.2 g/cc, significantly lower than Cu. The densities of CNT-Cu wires with various levels of internal filling along with Cu and starting CNT wires are provided in supplementary Table S1 for reference.

The resistivity of our fully filled MWCNT-Cu wires is still higher than previous single wall (SW) CNT-Cu composites with a similar continuous Cu matrix and CNT vol%^1–3^. Composites prepared by Subramaniam *et al*. with 45 vol% CNTs showed ρ_RT_ values only 30% higher than Cu^1, 2^ and the CNT-Cu through-silicon-vias prepared by Sun *et al*. performed nearly equivalent to Cu^3^. We attribute the dissimilar ρ_RT_ values to differences between the nanotubes present in our composite wires and those in the composites prepared by Subramaniam *et al*. and Sun *et al*. The microscale composite samples reported by Subramaniam *et al*. and Sun *et al*. contain aligned long SWCNTs running end-end between the contacts. On the other hand, our composite wires contain large-diameter MWCNTs with many nanotube ends between contacts. Further, the MWCNT bundles in our composite wires are twisted and not unidirectionally aligned along the wire axis.

### Resistance vs. temperature of MWCNT-Cu wires vs. internal Cu filling

The temperature vs. resistance behaviour (Fig. 3b) and TCR (Fig. 3b inset and supplementary Table S1) of the composite wires also change with the internal Cu filling, similar to ρ_RT_. The starting material MWCNT wires show resistance-decrease with temperature and a −ve TCR (~− 9.0 × 10^−4^/K). The TCR of CNT fibres can be + ve or −ve depending on CNT type, doping levels, etc^19, 27–32^. The resistance vs. temperature behaviour and TCR of our starting material MWCNT wires is agreement with trends and values reported for similar neat MWCNT assemblies spun from vertical arrays^19, 29–32^. With addition of Cu, the Cu-seeded wires show a less –ve TCR (~–4.0 × 10^−4^/K). This suggests that while MWCNTs still dominantly contribute to electron transport with temperature-increase, the metallic Cu seeds and outercoating also begin to participate, decreasing the –ve slope of the resistance vs. temperature plot. MWCNT-Cu wires with only Cu outercoating (no internal Cu filling) show metal-like resistance-increase with temperature and a + ve TCR of 3.0 × 10^−2^/K, close to that of Cu (3.3 × 10^−3^/K). This behaviour agrees with previous observations by Randeniya *et al*.^14^ Hence, in these samples, the dominant electron transport is through the continuous least resistive Cu outercoating. With increase in internal Cu filling, the slope is gradually suppressed and the TCR value drops to 2.8 × 10^−3^/K in the partially filled wires and finally to 1.7 × 10^−3^/K for the fully filled MWCNT-Cu composite wires. We see this halving of the TCR in the fully filled composite wires compared to Cu as a result of opposing resistance vs. temperature behaviours of Cu and MWCNTs. Therefore, in the fully filled MWCNT-Cu wires, although electron transport through the continuous metallic Cu matrix leads to an overall resistance-rise with temperature and a + ve TCR, the MWCNT bundles (which by themselves show a –ve TCR) lower the resistance-increase, reducing the TCR values for the composite.

The overall suppression in resistance-increase with temperature compared to Cu agrees with previous reports on CNT-Cu microscale samples and sheets^1, 3, 17^. However, the extent of TCR reduction seems to vary depending on CNT vol%, type, length, orientation, etc. in the composite. The lowest TCR we observe in our fully filled composite wires with ~40–45 vol% MWCNTs (½ that of Cu) is similar to values achieved by Sun *et al*.^3^ in aligned composite microstructures consisting of 40–50 vol% aligned several-100-μ-long SWCNTs running end to end. Shuai *et al*.^17^ report TCR values 80% of Cu in superaligned MWCNT-Cu composites due to a low 1 vol% CNT content. In contrast, Subramaniam *et al*.^1^ report an order of magnitude reduction in TCR compared to Cu for composites consisting of 45 vol% aligned 500–700 μm SWCNTs.

We highlight that the combination of ρ_RT_ similar to Cu and lower TCR than Cu leads to high-temperature resistivity values lower than Cu.^1, 3^ Despite a TCR ½ that of Cu, our composite wires with ρ_RT_ 10 × Cu show high-temperature ρ values higher than Cu. Therefore, improving our composite wires to achieve ρ_RT_ comparable to Cu combined with TCR values <Cu is vital for their future application as Cu wire replacements in high-temperature applications. We also note that we have discussed the MWCNT-Cu wire temperature dependence of electrical resistance solely based on contributions from MWCNTs and Cu. The MWCNT/Cu interface is bound to play a critical role in the electron transport. Further work is underway to characterize the CNT/Cu interface and identify its contribution to the electron transport in CNT-Cu composites.

### Current carrying capacity (CCC) of MWCNT-Cu wires vs. internal Cu filling

The ability of conducting wires to carry high currents without damage and withstand current surges is critical for their practical application in mainstream circuits. This capability is quantified by the CCC, which we measure as the maximum current density at which a given wire breaks in vacuum (at ~10^−3^ Pa). Wires break because of a combination of current-induced atomic diffusion and resistive Joule heating effects, together termed as electromigration^33^. Diffusion-dominated failure is more pronounced in loosely bonded metallic systems like Cu wires. CNT wires, however, fail mainly on account of Joule heating attributed to their high resistances caused by the presence of many tube-tube junctions^1, 34^. Previous studies have shown that the presence of CNTs in a Cu matrix can suppress surface and grain-boundary Cu diffusion^1, 17^. On the other hand, the presence of metallic Cu in CNT wires reduces resistance and thereby, Joule heating. Therefore, combining CNTs and Cu in wires can be expected to lead to increased current carrying capacities.

As shown in Fig. 4a, the fully filled composite wires with their homogeneous Cu and MWCNT distribution show high vacuum CCCs, slightly exceeding that of Cu (by ~28%). Our observation is analogous to the higher CCCs of microscale continuous Cu-matrix-CNT (45 vol%) composites than neat Cu previously observed.^1^ This high CNT-Cu CCC (>Cu) was attributed to CNTs suppressing Cu diffusion by increasing Cu diffusion activation energy (to ~2.0 eV vs. 0.6–1.0 eV in neat Cu systems).^1^ We expect MWCNTs in our fully filled composite wires to exert a similar effect in curbing Cu grain diffusion. The Cu-seeded wires and samples with partial and no internal Cu filling show low vacuum CCC values of ~1–2 × 10^4^ A/cm^2^, similar to the neat MWCNT wires. This is caused by disconnected smaller Cu grains within and the Cu outercoating not in contact with the MWCNTs, which can readily electromigrate, leading to failure at lower currents.

**Figure 7:**
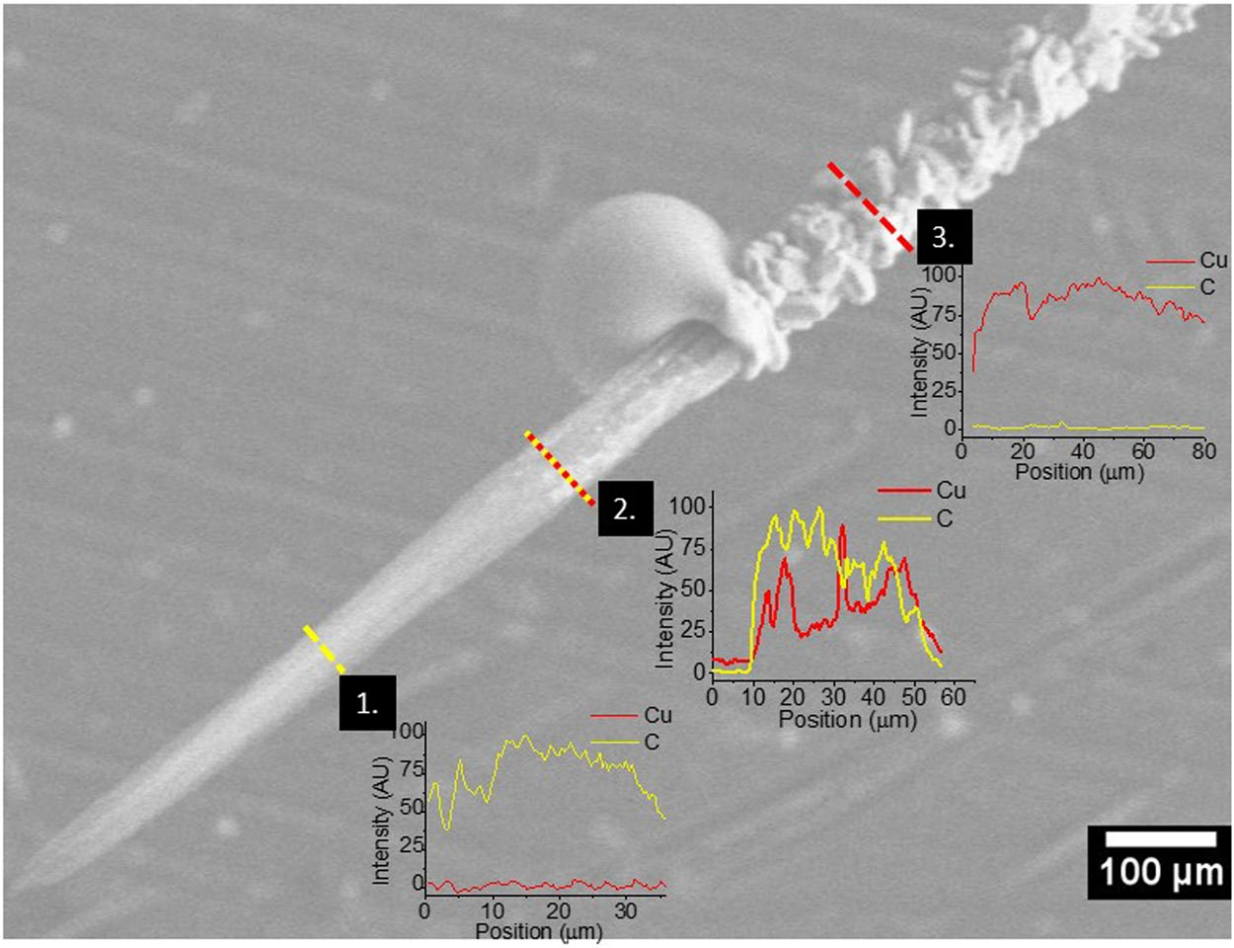
SEM image of the broken end of the MWCNT-Cu wire after CCC testing. The Cu and C EDX profiles along lines 1, 2, and 3 are also shown.

We note that the origin of current-induced failure in the MWCNT-Cu wires and MWCNT wires are dissimilar. A typical broken wire-end after CCC testing in vacuum of a fully filled MWCNT-Cu wire is shown in Fig. 7. The end consists of the CNT wire tail and a receded Cu coating forming a globule. The EDX line profiles of Cu and C at various points along the end are also shown. Only C signal from MWCNTs is observed near the breaking point and nearly no Cu is detected. Portions away from the breaking point show increased presence of Cu. The tested wire samples broke at around the midpoint and both broken ends showed the above described features. Further, all composite wires, irrespective of the internal Cu filling level showed similar broken ends after vacuum CCC testing. We conjecture that the MWCNT-Cu wire failure occurs predominantly by Cu diffusion followed by burn-out of the remaining MWCNT network. This is supported by the resistance-increase with applied current until break shown by all the Cu-containing wires (Fig. 4b) indicating that the dominant current transport is through Cu in the composite wires. The Cu diffusion may be induced by both the current flow and melting by Joule-heating. However, the neat MWCNT wires seem to fail primarily due to Joule effects i.e., heating up of the wires with increase in applied current. The heating up is evidenced from the resistance reduction of the MWCNT wires with applied current (Fig. 4c).

We note that the CCC of CNT-Cu composites measured in this work cannot be compared with values reported in other studies^1, 17^ because of differences in the measurement set-up and sample configuration. Measured CCC values depend on the sample environment (vacuum/inert gas), current application rate and dwell time, presence of a substrate in contact with samples, sample size, etc.^17, 34^ These experimental factors affect the sample’s chemical state and dissipation of heat generated by Joule effects and thereby, the electromigration-driven failure. For instance, both Cu and MWCNT-Cu wires showed a ~30% decrease in CCCs when exposed to laboratory atmosphere (10^5^ Pa) during measurement (Supplementary Fig. S3) due to sample oxidation. To demonstrate the effect of sample configuration: while our freestanding MWCNT-Cu wires without any substrate to aid heat dissipation show maximum vacuum CCCs of ~6.3 × 10^4^ A/cm^2^, CNT-Cu microscopic lines on Si/Si_3_N_4_ substrates show vacuum CCC values in the order of 10^8^ A/cm^2^.

In agreement with literature^1, 17^, our results do demonstrate the benefit of the presence of CNTs in a continuous Cu matrix to achieve CCCs higher than Cu (when measured under identical conditions). However, the extent of CCC increase in CNT-Cu relative to Cu varies in the literature. The vacuum CCC of our fully filled MWCNT-Cu wires is ~6.3 × 10^4^ A/cm^2^ compared to ~4.9 × 10^4^ A/cm^2^ shown by Cu wires of comparable dimensions under identical measurement conditions. Shuai *et al*.^17^ report a CCC of 1.2 × 10^4^ A/cm^2^ for 1 vol% superaligned MWCNT-Cu composites compared to 8.8 × 10^3^ A/cm^2^ for Cu (measured under Ar flow), which is a 36% increase. Contrastingly, Subramanian *et al*. report a 100-fold increase in CCC (measured at 10^−4^ Pa) over Cu for their microscale aligned 45 vol% SWCNT-Cu composites with nanotubes running from end to end^1^. Hence, similar to electrical resistivity and TCR, the composite composition, nanotube type, alignment, etc. also affect the stability to current of CNT-Cu. Further, as in the case of Shuai *et al*.^17^, our MWCNT-Cu wire CCC test samples were long (~1 cm) and includes multiple MWCNT ends and junctions, which are expected to negatively affect the CNT-Cu CCC values.

## Conclusions

We report conducting MWCNT-Cu composite wires with 2/3^rd^ the density of Cu containing nanotubes distributed uniformly throughout a continuous Cu matrix all along the wire length. Up to 10 cm long wires of such MWCNT-Cu composites could be fabricated by 2-step Cu electrodeposition into industrial nanotube wire spools, demonstrating potential fabrication scalability. The 2-step electrodeposition facilitated Cu penetration within the MWCNT wires, leading to the formation of the continuous Cu matrix. We measured the essential electrical properties of these MWCNT-Cu wires, including ρ_RT_, resistance-change with temperature, and CCC in vacuum. We also systematically varied the Cu spatial distribution in the composite wire surface and bulk and correlated associated changes in the overall electrical properties. Our studies indicate that maximizing internal Cu filling throughout and achieving a continuous Cu matrix is crucial to achieving favourable overall electrical performance. With potential fabrication scalability, low density, and favourable electrical performance of low ρ_RT_, lower resistance-change with temperature than Cu, and high CCC in vacuum slightly exceeding that of Cu, our composite wires show promise as lightweight alternatives to Cu wires. A comparison of the electrical performance of our MWCNT-Cu wires with previous similar CNT-Cu composites suggests that the CNT structure and alignment may affect the composite performance.

## Methods

The MWCNT-Cu wires were prepared by 2-step galvanostatic electrodeposition (Fig. 1a) of Cu into industrial MWCNT wires available as 10 m spools (Muratec, Murata Machinery Ltd., Japan). The starting MWCNT wires (diameter ~40 μm, supplementary Fig S4) were neat single filaments manufactured by continuous twist-spinning of substrate-grown vertical MWCNT arrays (CNT diameter ~20 nm, array height ~600 μm, CNT content >95 wt%). Two-step Cu deposition into MWCNT wires was carried out on a VMP3 electrochemical workstation (Princeton applied research). A three-electrode set-up consisting of two Cu anodes and the MWCNT wire mounted on a stainless steel mesh cathode was used. In our current experimental set-up, CNT wire lengths ranging from 1.5 cm to 10 cm could be mounted on the cathode. The electrolytes for the initial Cu seeding (step 1) and subsequent seed growth for filling (step 2) were anhydrous copper acetate (Sigma Aldrich) in acetonitrile (Wako pure chemicals) and commercial aqueous acidified CuSO_4_ solution (ATMI, without accelerators/suppressors), respectively. Various electrodeposition parameters of the two steps were optimized to achieve uniform internal Cu seeding and maximum final internal Cu filling (or full filling), as judged by internal structure characterization. The optimum current densities for the seeding and filling steps were 2.5 mA/cm^2^ and 10 mA/cm^2^, respectively. After each deposition step, reductive annealing under continuous H_2_ flow (250 sccm, 3 h) was carried out to remove CuO^1^. To study the effect of Cu spatial distribution on MWCNT-Cu wire performance, in addition to wires with Cu-seeding and full filling, process conditions were tuned to obtain wires with partial Cu filling and samples with only Cu outercoating and no internal Cu filling (core-sheath structures). The samples with only Cu outercoating were prepared by direct electrodeposition of the MWCNT wires in aqueous CuSO_4_ solution without prior Cu seeding. Wires with partial Cu internal filling were obtained by subjecting the Cu-seeded wires to aqueous deposition at lower a current density of 2 mA/cm^2^.

The diameter of the various wire samples was measured by optical microscopy. The internal structure and Cu spatial distribution in the various types of MWCNT-Cu wires was obtained by characterizing multiple cross sections in a scanning electron microscope (SEM, Hitachi S4800) attached with an energy-dispersive X-ray spectroscope (EDX, Bruker). The total Cu content was obtained by weights measured at various stages of deposition using a high-precision balance (Mettler Toledo UMX2, resolution = 0.1 μg). The internal Cu filling wt% was calculated by subtracting the amount of Cu in the outercoating from the total Cu content. The Cu content in the outercoating was evaluated from the coating thickness using cross section SEM (CS-SEM) images.

The electrical performance, i.e., ρ_RT_, resistance vs. temperature behaviour, and CCC of the MWCNT-Cu wires with various internal Cu filling levels was measured. The corresponding properties of the starting neat MWCNT wires and pure Cu wires were also measured under identical conditions for comparison. The electrical resistance values were acquired on a 4-probe set-up (Lakeshore PS-100 probe station, Keysight B1500A analyser) with the test wires mounted on sputter-deposited Au pads (Fig. 3a, inset). Resistance values above room temperature were obtained in vacuum (10^−3^ Pa) to avoid Cu oxidation. The sample temperature was regulated by a heating stage in the probe station using Lakeshore Model 236 controller. The CCC of the CNT-Cu wires was measured on a home-built set-up (Fig. 4a, inset). The test wires were freestanding (not in contact with any substrate) and suspended across a pair of electrodes supplying current. The current was increased in steps of 1 mA with a dwell time of 1 s until samples failed around the midpoint. The current application rate was maintained constant for all wires tested. All experiments were carried out in vacuum (10^−3^ Pa). The CCC was calculated as the breaking current density. For comparison, the CCC of Cu wires of comparable dimensions to the CNT and CNT-Cu test wires (diameter ~50 μm and 100 μm) were also recorded. The resistance vs. applied current was plotted for all wire samples.

## Electronic supplementary material

Supplementary figures S1-S4 and supplementary table S1
