# Supplementary material for: Electrical performance of lightweight CNT-Cu composite wires impacted by surface and internal Cu spatial distribution
Source: Sci Rep. 2017 Aug 24;7:9267. doi: 10.1038/s41598-017-09279-x (PMC5570951; doi:10.1038/s41598-017-09279-x)
Supplement: Supplementary file 1 — Supplementary figures S1-S4 and supplementary table S1 [file 41598_2017_9279_MOESM1_ESM.doc]

# Electrical performance of lightweight CNT-Cu composite wires impacted by surface and internal Cu spatial distribution

Rajyashree Sundaram^1^, Takeo Yamada^1,2^, Kenji Hata^1,2^, Atsuko Sekiguchi^1,2^*

*Affiliations:*

**^1^Technology Research Association for Single Wall Carbon Nanotubes (TASC), Central 5, 1-1-1 Higashi, Tsukuba 305-8565, Japan**

**^2^National Institute of Advanced Industrial Science and Technology (AIST), Central 5, 1-1-1 Higashi, Tsukuba 305-8565, Japan**

*Corresponding author: E-mail: [atsuko-sekiguchi@aist.go.jp](mailto:atsuko-sekiguchi@aist.go.jp)

Present address: Rajyashree Sundaram

**National Institute of Advanced Industrial Science and Technology (AIST), Central 5, 1-1-1 Higashi, Tsukuba 305-8565, Japan**

## Supplementary Information

Figure S1. Multiple cross sections (CS) along the length (1 cm) of a fully filled MWCNT-Cu wire. The dotted ellipsoids represent the cross section outlines.


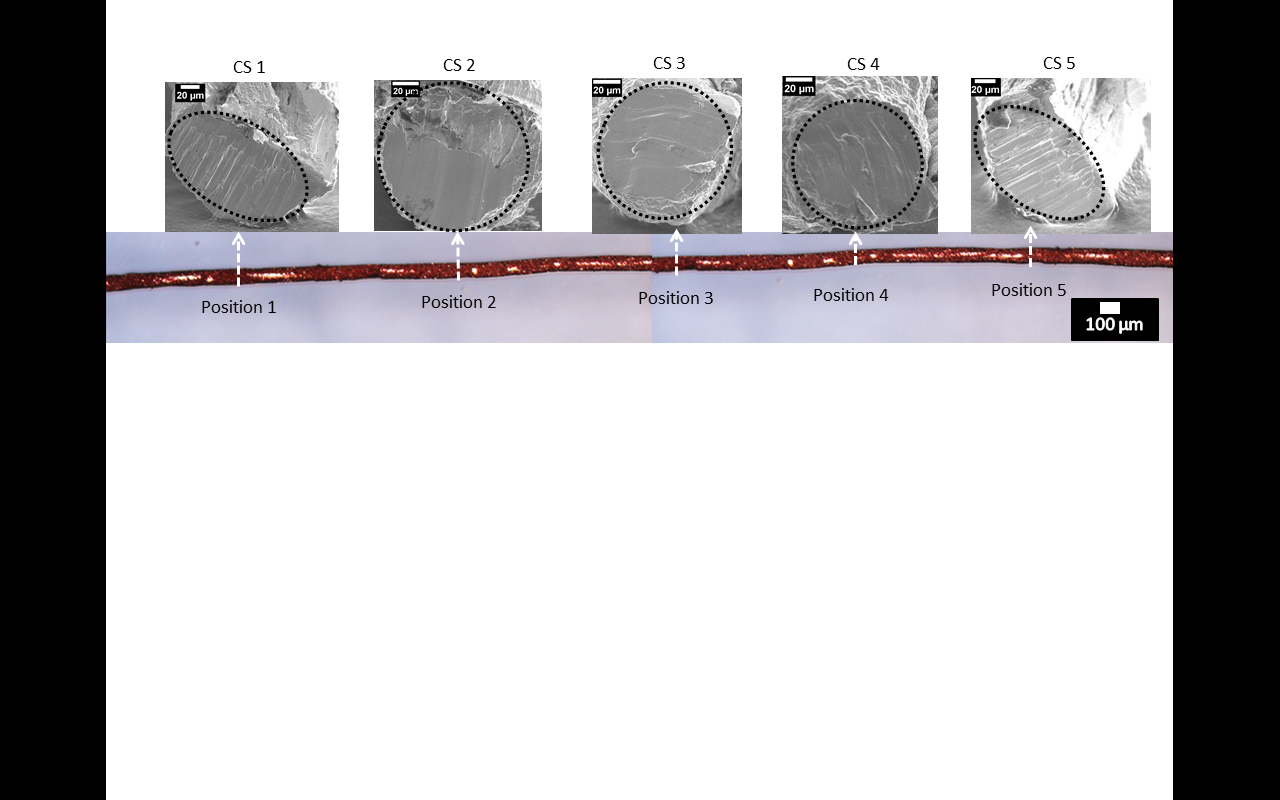


Figure S2. Internal Cu content vs. total Cu content of MWCNT-Cu wires

Figure S3. Comparison of CCCs of fully filled MWCNT-Cu composite and Cu wires in vacuum and air.

Figure S4. a. SEM images and b. thermogravimetry and differential thermogravimetry data of the industrial array-spun MWCNT wires used for making MWCNT-Cu composite wires

**a.**


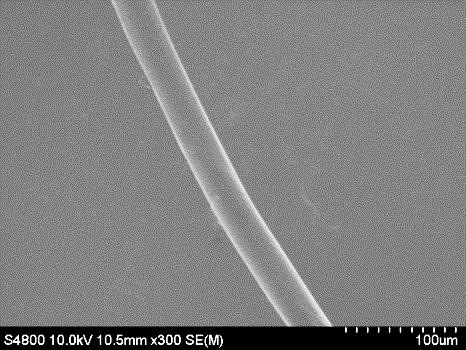

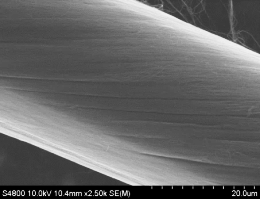


Wire alignment

**Twist**


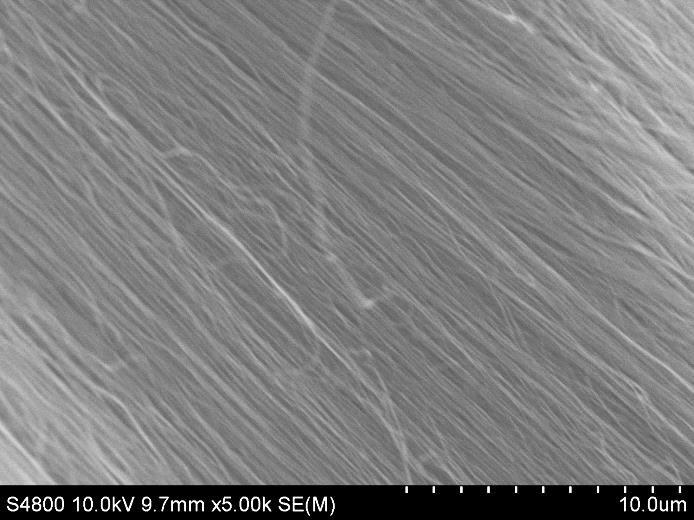


**CNT alignment along twist**

**b.**

Wt loss at 400 deg C ~ 4 wt%

0% residue

**CNT content ~ 96 wt%**

Table S1. Density, room temperature resistivity, and TCR values of various wire samples

| **Wire sample** | **Density (g/cc)** | **Resistivity (ohm cm)** | **TCR (/K)** |
| --- | --- | --- | --- |
| **Starting MWCNT** | 0.55 ± 0.13 | 1.1 × 10^-3^ ± 1.0 × 10^-4^ | -9.0 × 10^-4^ ± 8.2 × 10^-5^ |
| **Cu-seeded MWCNT** | 3.2 ± 0.27 | 7.5 × 10^-5^ ± 1.0 × 10^-5^ | -4.0 × 10^-4^ ± 6.3 × 10^-5^ |
| **Fully filled MWCNT-Cu** | 5.2 ± 0.25 | 1.6 × 10^-5^ ± 1.6 × 10^-6^ | 1.7 × 10^-3^ ± 1.9 × 10^-4^ |
| **Partially filled MWCNT-Cu** | 3.4 ± 0.31 | 3.1 × 10^-5^ ± 3.3 × 10^-6^ | 2.8 × 10^-3^ ± 3.5 × 10^-4^ |
| **No filling MWCNT-Cu** | 1.6 ± 0.28 | 3.3 × 10^-5^ ± 2.7 × 10^-6^ | 3.0 × 10^-3^ ± 4.1 × 10^-4^ |
| **Cu** | 8.9 | 1.6 × 10^-6^ ± 1.8 × 10^-7^ | 3.3 × 10^-3^ ± 4.3 × 10^-4^ |

To note: The sample densities were calculated from mass values measured in a high-precision balance and diameters measured by optical microscopy assuming circular cross sections.
